# Absence of association between angiotensin converting enzyme polymorphism and development of adult respiratory distress syndrome in patients with severe acute respiratory syndrome: a case control study

**DOI:** 10.1186/1471-2334-5-26

**Published:** 2005-04-09

**Authors:** KC Allen Chan, Nelson LS Tang, David SC Hui, Grace TY Chung, Alan KL Wu, Stephen SC Chim, Rossa WK Chiu, Nelson Lee, KW Choi, YM Sung, Paul KS Chan, YK Tong, ST Lai, WC Yu, Owen Tsang, YM Dennis Lo

**Affiliations:** 1 The Centre for Emerging Infectious Diseases, The Chinese University of Hong Kong, Prince of Wales Hospital, Hong Kong; 2 Department of Chemical Pathology, The Chinese University of Hong Kong, Prince of Wales Hospital, Hong Kong; 3 Department of Medicine and Therapeutics, The Chinese University of Hong Kong, Prince of Wales Hospital, Hong Kong; 4 Department of Microbiology, The Chinese University of Hong Kong, Prince of Wales Hospital, Hong Kong; 5 Department of Medicine and Geriatrics, Princess Margaret Hospital, Hong Kong

## Abstract

**Background:**

It has been postulated that genetic predisposition may influence the susceptibility to SARS-coronavirus infection and disease outcomes. A recent study has suggested that the deletion allele (D allele) of the *angiotensin converting enzyme *(*ACE*) gene is associated with hypoxemia in SARS patients. Moreover, the *ACE *D allele has been shown to be more prevalent in patients suffering from adult respiratory distress syndrome (ARDS) in a previous study. Thus, we have investigated the association between ACE insertion/deletion (I/D) polymorphism and the progression to ARDS or requirement of intensive care in SARS patients.

**Method:**

One hundred and forty genetically unrelated Chinese SARS patients and 326 healthy volunteers were recruited. The ACE I/D genotypes were determined by polymerase chain reaction and agarose gel electrophoresis.

**Results:**

There is no significant difference in the genotypic distributions and the allelic frequencies of the ACE I/D polymorphism between the SARS patients and the healthy control subjects. Moreover, there is also no evidence that ACE I/D polymorphism is associated with the progression to ARDS or the requirement of intensive care in the SARS patients. In multivariate logistic analysis, age is the only factor associated with the development of ARDS while age and male sex are independent factors associated with the requirement of intensive care.

**Conclusion:**

The ACE I/D polymorphism is not directly related to increased susceptibility to SARS-coronavirus infection and is not associated with poor outcomes after SARS-coronavirus infection.

## Background

The outbreak of the severe acute respiratory syndrome (SARS) has made a great impact to the health care systems around the world. The pandemic affected over 8000 individuals and resulted in 774 deaths worldwide [1]. Several clinical parameters, including male sex [2,3], age of over 60 years [2,3], elevated lactate dehydrogenase activity [2-4], low platelet count [2] and high viral load on presentation [5], have been identified to be predictive of the severity of the disease in affected individuals. Moreover, it has been postulated that genetic variations of the host and the virus may account for the individual difference in the susceptibility to the infection and the severity of the disease. With regard to viral factors, it has been shown that there is no significant difference in the genetic sequences of viruses causing the two major outbreaks in Hong Kong, namely the Prince of Wales Hospital and Amoy Gardens outbreaks, despite the significant difference in the mortality rates and diarrheal rates of the two cohorts [6]. Furthermore, several association studies have been conducted to investigate the possible contribution of host genetic factors in the determination of the susceptibility and prognosis of SARS-coronavirus infection. Thus, certain human leukocyte antigen subtypes have been shown to be more prevalent in SARS patients [7] and in those who had poorer outcomes [8]. On the other hand, the polymorphism in the angiotensin converting enzyme II gene, coding for a functional receptor of the SARS-coronavirus, is not associated with the susceptibility or outcome of SARS [9]. Recently, it has also been reported that the deletion of the 287 bp *Alu *repeat (D allele) in intron 16 of the *ACE *gene is associated with hypoxemia in SARS patients [10]. However, there are several limitations to this previous study. First, only 44 SARS patients were studied. Second, hypoxemia was arbitrarily defined as requiring oxygen supplementation. Moreover, patients who died were excluded from the study. These factors may be potential confounders to a genetic association study.

Therefore, in this study, we investigated the association of the *ACE *insertion/deletion (I/D) polymorphism of the 287 bp *Alu *repeat to the susceptibility to SARS and the development of adult respiratory distress syndrome (ARDS) with a larger population.

## Methods

### Study population

This study was reviewed and approved by the Ethical Committee of the Prince of Wales Hospital, Hong Kong. Patients who were admitted to the hospitals of the New Territories East cluster of Hong Kong for the treatment of SARS were recruited retrospectively. The recruitment of patients depended on the availability of blood samples. All patients, including survivors and deceased patients, with available blood samples were recruited. For genetically related SARS patients, only the index case (the first individual who developed symptoms) was recruited. All patients were of Chinese ethnicity and fulfilled the World Health Organisation case definition of probable SARS [11]. Three hundred and twenty-six healthy individuals undergoing routine health check were recruited as controls. The control subjects were recruited before the SARS epidemic and none of them had respiratory symptoms. All control subjects were ethnical Chinese and were not genetically related.

### Definition of adult respiratory distress syndrome (ARDS) and patients with severe disease

The association between genotype and disease outcome was studied in the SARS patients. Two categories of patients were considered as having a severe disease: (1) patients who developed ARDS; and (2) patients who required admission to the intensive care unit (ICU). A patient was classified as having ARDS if he or she fulfilled all criteria of the joint American/European Consensus for ARDS [12], including: (1) acute onset of respiratory distress; (2) presence of bilateral infiltrates on chest X-ray; (3) having a ratio of arterial partial pressure of oxygen to inspired fractional oxygen concentration (PaO_2_/FiO_2_) of less than 26.8 kPa and absence of clinical evidence of left heart failure.

### ACE Genotyping

DNA was extracted from whole blood using a QIAamp DNA Blood Mini Kit (Qiagen) with the 'Blood and Body Fluid Spin protocol' as recommended by the manufacturer. ACE I/D genotypes were determined by polymerase chain reaction amplification. The forward and reverse primers were 5'-CTGGAGACCACTCCCATCCTTTCT-3' and 5'-GATGTGGCCATCACATTCGTCAGAT-3', respectively. Reactions were set up in a volume of 25 μl containing 0.1 μM of each primer, 1X buffer II (Applied Biosystems), 2 mM MgCl_2_, 0.2 mM of each dNTP, 1.25 U Taq polymerase (AmpliTaq Gold DNA polymerase, Applied Biosystems) and 20 ng DNA. After initial denaturation at 95°C for 12 min, the reaction mixtures were subjected to 40 cycles of 94°C for 1 min, 58°C for 1 min and 72°C for 1 min, and a final extension at 72°C for 5 min. This method yielded amplification products of 480 bp for the I allele and 192 bp for the D allele. The products were electrophoresed and visualized in 2% agarose gels with ethidium bromide.

### Statistical analysis

Statistical analyses were performed using SigmaStat, Ver. 3.0; SPSS. Disease associations were compared by chi-square tests. Univariate and multivariate logistic regression analyses were performed to identify predictors of ARDS or the outcome of SARS.

## Results

### Demographics

One hundred and forty SARS patients (67 males, 73 females) and 326 healthy individuals (172 males, 154 females) were recruited. The mean ages of the SARS patients and control subjects were 39.9 and 42.5 years, respectively (*p *= 0.93). Seventeen of the 140 SARS patients developed ARDS during the course of their illness. The demographic data of the SARS patients who had or had not developed ARDS are summarized in table 1. Patients who developed ARDS were significantly older than those who did not develop ARDS (p < 0.001). There was no significant difference in gender, smoking habits, hepatitis B status and the presence of comorbidity between the two groups. Thirty-five patients required intensive care and sixteen died. Patients who required intensive care were significantly older than those with milder disease.

**Table 1:** Demographic data of SARS patients. The patients are categorized by the development of ARDS (ARDS vs non-ARDS). All patients who had developed ARDS required intensive care. The numbers listed in the table represent the number of patients (except the numbers list in the first row which signify age).

	**ARDS**	**non-ARDS**
Age mean (range)	59.1 years (24–83)	36.9 years (21–76)
M:F	9/8	58/65
Smoking	2	5
HbsAg positive	0	7
Comorbidities		
chronic obstructive pulmonary disease	0	3
Ischemic heart disease	5	2
Cerebral vascular disease	3	4
Cancer	0	2
Diabetes mellitus	1	6
Chronic renal disease	0	1
Liver cirrhosis	0	1

### Genotypic and allelic frequencies of ACE I/D polymorphism

The genotypic distributions and allelic frequencies of ACE I/D polymorphism in the SARS patients and control subjects are shown in table 2. The genotypic distributions of the SARS patients and the healthy control subjects follow the Hardy-Weinberg equilibrium using chi-square analysis. There was no significant difference in the genotypic distributions (χ^2 ^value = 1.43, df = 2, *p *= 0.489) and allelic frequencies (χ^2 ^value = 0.000624, df = 1, *p *= 0.980) of the two groups. Among the SARS patients, we further analyzed the genotypic distributions and allelic frequencies of ACE I/D polymorphism in patients who developed ARDS and in those who did not develop ARDS in the course of their illness. The results are shown in table 3a. There was no significant difference in the genotypic distributions (χ^2 ^value = 4.361, df = 2, *p *= 0.113) and allelic frequencies (χ^2 ^value = 1.376, df = 1, *p *= 0.241) between the two groups. Besides, there was also no significant difference in the genotypic distributions (χ^2 ^value = 2.489, df = 2, *p *= 0.288) and allelic frequencies (χ^2 ^value = 1.424, df = 1, *p *= 0.233) between patients who did or did not require intensive care. The results are shown in table 3b.

**Table 2:** Genotypic and allelic frequencies of ACE I/D polymorphism in SARS patients and control subjects

	***SARS cases***	***Controls***
	n = 140	n = 326
*Genotypic frequency*		

***DD***	15 (11%)	43 (13%)
***ID***	65 (46%)	133 (41%)
***II***	60 (43%)	150 (46%)
χ^2 ^value = 1.43, df = 2, *p *= 0.489		
		
*Allelic frequency*		

***D***	95 (34%)	219 (34%)
***I***	185 (66%)	433 (66%)
χ^2 ^value = 0.000624, df = 1, *p *= 0.980		

**Table 3a:** Genotypic and allelic frequencies of ACE I/D polymorphism in SARS patients who had developed or had not developed ARDS

	***ARDS***	***Non-ARDS***
	n = 17	n = 123
*Genotypic frequency*		

***DD***	2 (12%)	13 (10%)
***ID***	4 (23%)	61 (50%)
***II***	11 (65%)	49 (40%)
χ^2 ^value = 4.361, df = 2, *p *= 0.113		
		
*Allelic frequency*		

***D***	8 (24%)	87 (35%)
***I***	26 (76%)	159 (65%)
χ^2 ^value = 1.376, df = 1, *p *= 0.241		

**Table 3b:** Genotypic and allelic frequencies of ACE I/D polymorphism in SARS patients who had or had not required intensive care (ICU vs non-ICU)

	***ICU***	***Non-ICU***
	n = 35	n = 105
*Genotypic frequency*		

DD	3 (9%)	12 (11%)
ID	13 (37%)	52 (50%)
II	19 (54%)	41 (39%)
χ^2 ^value = 2.489, df = 2, *p *= 0.288		
		
*Allelic frequency*		

D	19 (27%)	76 (36%)
I	51 (73%)	134 (64%)
χ^2 ^value = 1.424, df = 1, *p *= 0.233		

### Logistic regression on the development of ARDS or requirement of intensive care

In the univariate analysis, we did not detect any significant difference in the number of D alleles in the ACE polymorphism between patients who did and did not develop ARDS (p = 0.169, OR = 0.549 (95% CI: 0.23–1.29). Following multivariate logistic regression analysis, age was found to be the only significant factor that determined the development of ARDS in SARS patients (table 4a). In the multivariate analysis for the requirement of intensive care, we have shown that age and male sex are associated with the requirement of intensive care (table 4b).

**Table 4a:** Logistic regression on the development of ARDS in SARS patients

***Variables***	**Univariate analysis**		**Multivariate analysis**	
	
	***Odds ratio (95% CI)***	***p value***	***Odds ratio (95% CI)***	***p value***
ACE (no. of D allele)	0.64 (0.28 – 1.47)	0.30	0.74 (0.27 – 1.98)	0.46
Age	1.08 (1.04 – 1.12)	<0.01	1.10 (1.04 – 1.16)	<0.01
Male sex	1.26 (0.46 – 3.48)	0.65	0.22 (0.04 – 1.10)	0.06
Smoking	3.15 (0.56 – 17.67)	0.19	1.03 (0.11 – 9.65)	0.98
HbsAg	1 (0 – ∞)	0.99	1 (0.00 – ∞)	0.99
Co-morbidities	6.15 (2.11 – 17.96)	<0.01	1.61 (0.36 – 7.26)	0.53

**Table 4b:** Logistic regression on the requirement of intensive care in SARS patients

***Variables***	**Univariate analysis**		**Multivariate analysis**	
	
	***Odds ratio (95% CI)***	***p value***	***Odds ratio (95% CI)***	***p value***
ACE (no. of D allele)	0.69 (0.38 – 1.27)	0.23	0.77 (0.39 – 1.51)	0.46
Age	1.06 (1.03 – 1.08)	<0.01	1.06 (1.02 – 1.09)	<0.01
Male sex	4.50 (1.92 – 10.58)	<0.01	3.20 (1.25 – 8.19)	0.02
Smoking	2.37 (0.50 – 11.14)	0.28	0.68 (0.10 – 4.52)	0.69
HbsAg	2.37 (0.50 – 11.14)	0.28	2.73 (0.52 – 14.43)	0.24
Co-morbidities	6.15 (2.11 – 17.96)	<0.01	1.61 (0.36 – 7.26)	0.53

## Discussion

The possible contribution of host genetic factors to the susceptibility and outcome of SARS-coronavirus infection has been investigated through several association studies [7-10]. In contrast to a recent report showing an association between the presence of the D allele of the *ACE *gene and hypoxemia in SARS patients [10], we have shown that the I/D polymorphism of the *ACE *gene is associated with neither increased susceptibility to SARS-coronavirus infection nor progression to ARDS once infected. In multivariate logistic regression analysis, we have identified that age is the only significant factor associated with the development of ARDS while age and male sex are independently associated with the requirement of intensive care in SARS patients. Our findings are consistent with other published reports [2,3].

There are several possible explanations for the discrepancies in our conclusion and that by Itoyama et al [10] concerning the association between *ACE *polymorphism and the outcome of SARS. First, the inclusion of subjects within the same family and exclusion of deceased patients by the previous study might cause potential bias, especially when the frequency of the DD genotype was reported to be as low as 6% in control subjects [10]. In this study, we have only included the index patient if more than one member in a family developed SARS. Second, we have used a well defined endpoint of ARDS instead of the requirement of supplemental oxygen. SARS infection commonly leads to respiratory distress and over 80% of patients were given supplemental oxygen during the course of their illness in our cohort. Therefore, it seems to be more appropriate to use ARDS instead of the requirement of oxygen supplement to define the severity of SARS. As ARDS is the more severe end of the spectrum of disease progression, any potential association between genotype and disease progression would become even more obvious when the most severe cases were considered. Similarly, the disease outcome was not associated with ACE I/D genotype when we also used another broader definition for severe disease after SARS infection (requiring intensive care or death).

Previous studies on Caucasian populations have suggested that the presence of the D allele of the *ACE *gene is associated with increased incidence of ARDS [13]. This effect has been postulated to be related to the higher enzyme activity in individuals with DD genotype [15]. However, it is unclear whether these observations can also be seen in Chinese as the frequencies of DD genotype and D allele of the *ACE *gene are much lower in Chinese than in Caucasian subjects [13,16]. Furthermore, the SARS-coronavirus characteristically affects the pneumocytes, and the formation of multinucleated pneumocytes and intrabronchial fibrogranulation (bronchiolitis obliterans organizing pneumonia-like lesions) are commonly observed in the lung biopsies of SARS patients in addition to the typical pathological changes of ARDS [17]. Therefore, it is possible that the pathogenesis and genetic factors predisposing to SARS-related ARDS may be different from ARDS resulted from other respiratory illnesses.

Previous reports have highlighted the inconsistency of the results of genetic association studies for complex diseases [18,19]. This inconsistency may be attributable to the difference in the genetic composition of the studied population and study design. Here, we showed that both susceptibility and disease outcome of SARS infection were not associated with ACE I/D polymorphism among Chinese patients in contrast to the recent report studying Vietnamese patients [10]. The sample size was definitively larger in our study. Two different better-defined criteria were used as indicators of severe disease progression, yet no association was found between disease severity and ACE I/D genotype. The D allele which was the hypothetical high risk allele [13], did not show any sign of over-representation in the subgroups of patients with severe disease.

## Conclusion

Our analysis indicates that ACE I/D polymorphism is not directly related to poor outcomes after SARS-coronavirus infection in Chinese.

## List of abbreviations used

ACE: angiotensin converting enzyme

SARS: severe acute respiratory syndrome

ARDS: adult respiratory distress syndrome

ACE I/D polymorphism: angiotensin converting enzyme insertion/deletion polymorphism

## Competing interests

YMDL, KCAC, RWKC, SCCC and YKT have filed patent applications on aspects concerning the genomics and detection of the SARS-coronavirus.

## Authors' contributions

KCAC, NLST, GTYC, and YMDL have contributed in the preparation of the manuscript and the overall study design. RWKC, SSCC, YKT, PKSC and YMS have contributed in the data analysis and conducting the experiments. DSCH, AKLW, NL, KWC, PKSC, STL, WCY and OT have contributed in the collection and analysis of clinical data from the patients.

## Pre-publication history

The pre-publication history for this paper can be accessed here:


